# Identifying COVID-19 Infections From a Vaccinated Population Using Specific IgA Antibody Test

**DOI:** 10.3389/fimmu.2022.821218

**Published:** 2022-01-31

**Authors:** Zhangkai J. Cheng, Peiyan Zheng, Mingshan Xue, Youpeng Chen, Baoqing Sun

**Affiliations:** ^1^ Department of Allergy and Clinical Immunology, Guangzhou Institute of Respiratory Health, State Key Laboratory of Respiratory Disease, National Clinical Research Center of Respiratory Disease, First Affiliated Hospital of Guangzhou Medical University, Guangzhou, China; ^2^ KingMed Diagnostics and KingMed School of Laboratory Medicine, Guangzhou Medical University, Guangzhou, China

**Keywords:** SARS-CoV-2, vaccine, IgA, diagnosis, COVID-19

## Abstract

We analyzed the serum from COVID-19 patients and vaccinated subjects, and found that the specific IgA titer level could be used to assist COVID-19 diagnosis, especially in China.

Positive PCR test for SARS-CoV-2 is the gold standard for confirming COVID-19 infection, but false negatives do occur, at which time the SARS-CoV-2 specific antibody test can be utilized to assist in diagnosis. Before the mass adoption of vaccines, specific IgM antibody titers could be used to indicate a recent infection. With the now widespread availability of vaccinations, virus-specific antibodies have now become extensively available in human bodies. Therefore, detection of specific IgM antibodies and IgG antibodies to aid the diagnosis of SARS-CoV-2 has long been out of use. However, due to the differences in the mechanism of action, the viral infection should induce higher IgA than vaccines. Therefore, we hypothesize that specific IgA antibodies found in the serum can be employed to help in the detection of COVID-19 infections.

554 participants were enlisted as subjects in this study. The COVID-19 patients enrolled from January to April, 2020, and were confirmed by real-time PCR and hospitalized in the Guangzhou Eighth People’s Hospital and the First Affiliated Hospital of Guangzhou Medical University. According to the “Diagnosis and Treatment Protocol for Novel Coronavirus Pneumonia” published by the National Health Commission of China, the patients were grouped into 59 (80.8%) mild and 14 (19.2%) severe cases. Healthy volunteers with COVID-19 vaccination, 247 for mRNA (Pfizer-BioNTech), 131 for inactivated (Sinopharm BBIBP) and 103 for inactivated (CoronaVac) were enrolled from January to October, 2021. The statistics of enrolled participants are summarized in **Supplementary Table S1**. The vaccine participants had not been exposed to SARS-CoV-2 infection before or throughout the study, due to the zero-COVID strategy employed in China (1). They were injected homologously with a two-shot regiment (28 days apart) for their respective vaccines. SARS-CoV-2-specific IgA, IgM and IgG were detected by the indirect chemiluminescence method (Guangzhou Kangrun Biotech Co., Ltd.^®^, Guangzhou, China). Geometric mean titer (GMT) was calculated using the geometric mean of the titer levels, and their 95% confidence interval (CI) was calculated with the Student’s t distribution on log-transformed data and then back transformed. Comparison between titer level differences between two cohorts was performed using Man-Whitney Wilcoxon Test. All figures were produced using MATLAB^®^ R2021a (Natick, MA, USA).

When specific antibodies IgA, IgM and IgG levels were compared between virus infection and vaccination, IgA was found to have the most significant difference (see **Supplementary S2** for IgM and IgG results). **Figure 1A** shows IgA levels at each time point. **Figure 1B** shows the distribution of SARS-CoV-2-specific IgA antibodies. The geometric mean titer (GMT) of IgA level in Pfizer-BioNTech vaccine cohort was 0.81, with a positive (titer≥1) rate of 45%; Sinopharm vaccine cohort was 0.17, with a positive rate of 2.2%; CoronaVac cohort was 0.23, with a positive rate of 4.2%; mild patients cohort were 1.7, with a positive rate of 48.1%; severe patients cohort were as high as 17.4, with 100% positive rate. IgA levels in the patient cohort were significantly higher than IgA levels in the vaccine cohort (*P*<0.05, all vaccinated vs. all infected). The level of IgA in mild patients started low at the beginning of infection detection, and rose after a few days into the infection.

**Figure 1:**
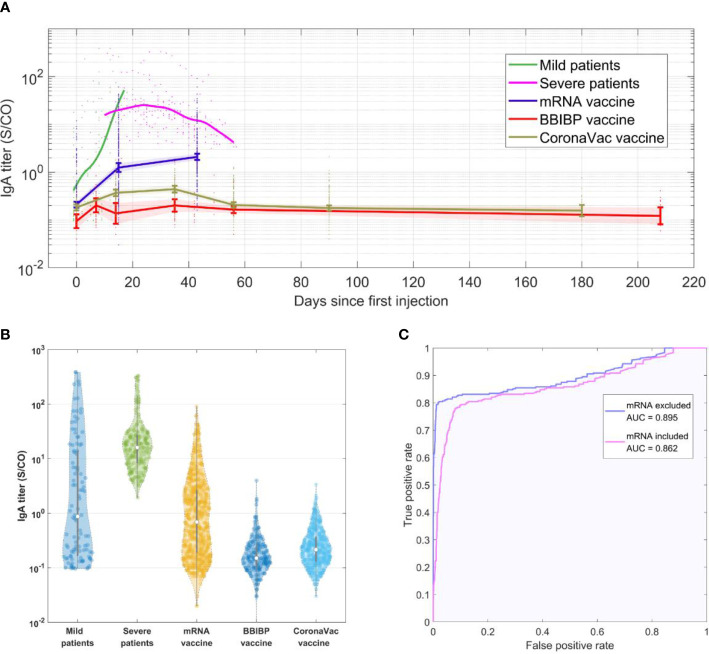
**(A)** SARS-CoV-2-IgA titer in each cohort over time. **(B)** Violin plot showing distribution of SARS-CoV-2-IgA titer levels in each cohort. The samples at different time points for each cohort were collected as a whole group. The gray lines in the center of each violin show the median, and the 25^th^ and 75^th^ percentiles. **(C)** ROC curve showing performance of diagnosing COVID-19 infection using SARS-CoV-2-IgA titer level.

Infected patients were found to have much greater IgA levels than vaccinated people after being infected for the same number of days as the vaccination. The SARS-CoV-2-specific antibodies in humans are likely to be produced in response to vaccination or infection, but an important difference between the two is that during virus infections, the main infection sites receive long-term high levels of stimulation, which induces strong mucosal immune responses and is reflected in the rise in IgA levels. This is consistent with multiple previous studies, which have found that the positive rate of specific IgA in a COVID-19 infection can reach over 50% within a week of confirmed infection (2, 3), with specific IgA titer levels peaking in the third week and the positive rate reaching 100% (2, 4). The IgA antibody levels were found to continue to remain high (within 42 days of onset) (2, 3, 5). Furthermore, according to our analysis, the specific IgA levels of the vaccine cohort were lower than the minimum IgA level of the severe patient cohort in more than 68% of mRNA vaccine cohort, and more than 95% of inactivated vaccine cohort. In addition, we used the data to create a receiver operating characteristic (ROC) curve of IgA antibody to determine SARS-CoV-2, regenerating an area-under-the-curve (AUC) of 0.862 (**Figure 1C**), which further verified our hypothesis. The accuracy is further increased when excluding subjects who have received mRNA vaccines, resulting in an AUC of 0.895.

Our results show that using specific-IgA titer levels to diagnose COVID-19 infection has a high prediction value, especially in a population where most of the population is vaccinated with inactivated vaccines, such as China. For most other vaccines, anti-nucleocapsid antibodies can be used as a tool to distinguish infected from vaccinated individuals, and should have a higher specificity compared to IgA titers (6). However, this is not the case for inactivated vaccines, as these should also induce antibodies against the nucleocapsid protein. As a result, we conclude that using the SARS-CoV-2-IgA antibody as a differential diagnosis of infection has a certain clinical reference significance.

## Data Availability Statement

The data that support the findings of this study are available upon reasonable request from the corresponding author.

## Ethics Statement

The studies involving human participants were reviewed and approved by First Affiliated Hospital of Guangzhou Medical University Ethical Committee. The patients/participants provided their written informed consent to participate in this study.

## Author Contributions

All authors made substantial contributions to the writing of this review, revised it critically for important intellectual content, approved the version to be published, and agreed to be accountable for all aspects of this review. All authors agreed with the content of this review and gave explicit consent to submit.

## Funding

This study was supported by Zhongnanshan Medical Foundation of Guangdong Province (ZNSA-2021005, ZNSA-2020001, ZNSA-2021016), State Key Laboratory of Respiratory Disease, Guangdong-Hong Kong-Macao Joint Laboratory of Respiratory Infectious Disease (GHMJLRID-Z-202102), Emergency key project of Guangzhou Laboratory (EKPG21-30-2), Cultivation Project of the First Affiliated Hospital of Guangzhou Medical University (ZH202105).

## Conflict of Interest

The authors declare that the research was conducted in the absence of any commercial or financial relationships that could be construed as a potential conflict of interest.

## Publisher’s Note

All claims expressed in this article are solely those of the authors and do not necessarily represent those of their affiliated organizations, or those of the publisher, the editors and the reviewers. Any product that may be evaluated in this article, or claim that may be made by its manufacturer, is not guaranteed or endorsed by the publisher.
