# Supplementary material for: Identifying COVID-19 Infections From a Vaccinated Population Using Specific IgA Antibody Test
Source: Front Immunol. 2022 Jan 31;13:821218. doi: 10.3389/fimmu.2022.821218 (PMC8841746; doi:10.3389/fimmu.2022.821218)

**Supplementary S2.** Results for SARS-CoV-2-specific IgM and IgG. (A) Violin plot showing distribution of SARS-CoV-2-IgM titer levels in each cohort. (B) SARS-CoV-2-IgM titer in each cohort over time. (C) Violin plot showing distribution of SARS-CoV-2-IgG titer levels in each cohort. (D) SARS-CoV-2-IgG titer in each cohort over time.

A
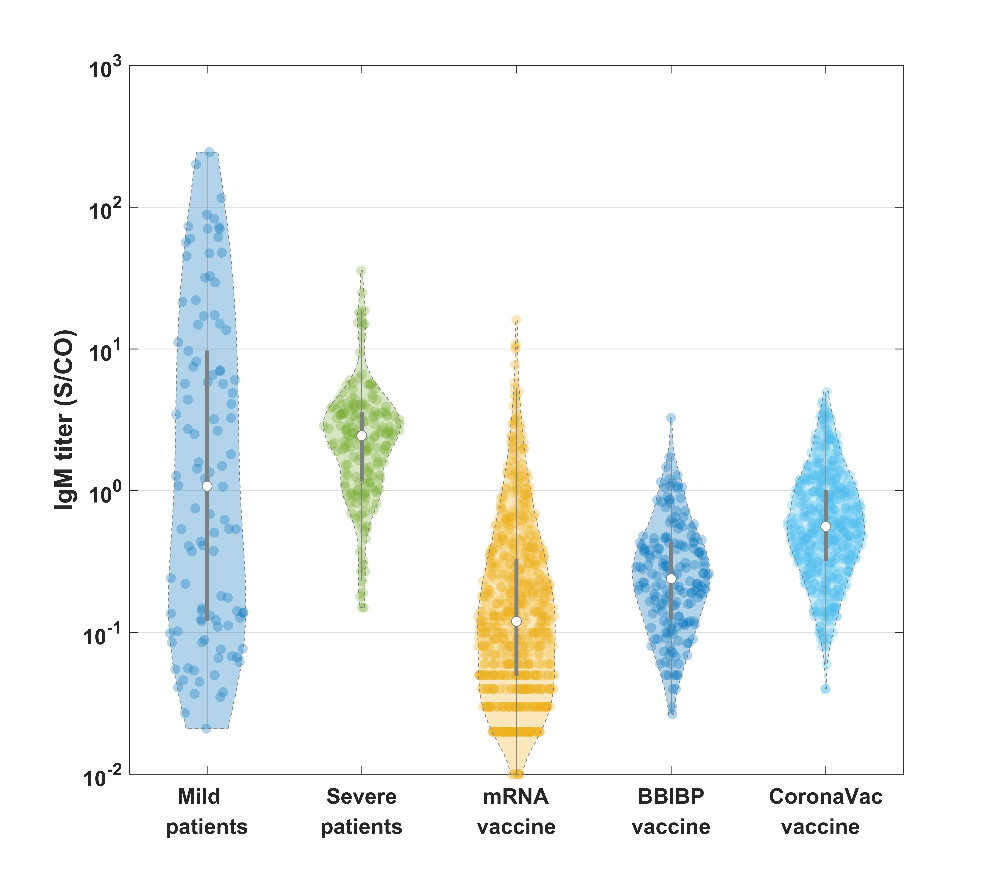


B
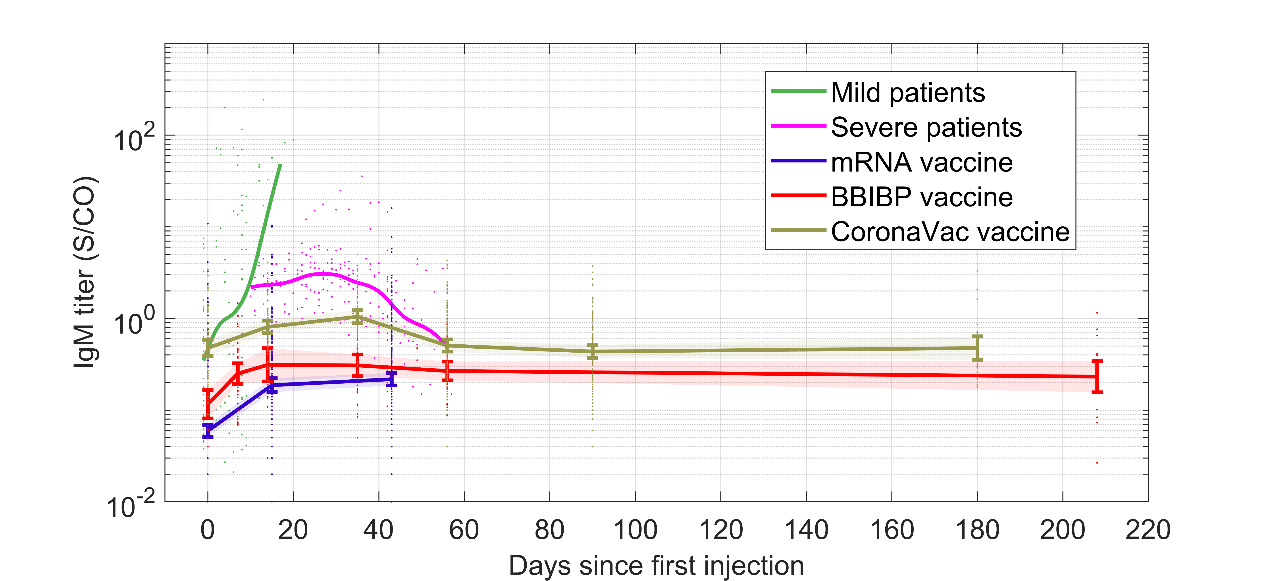


C
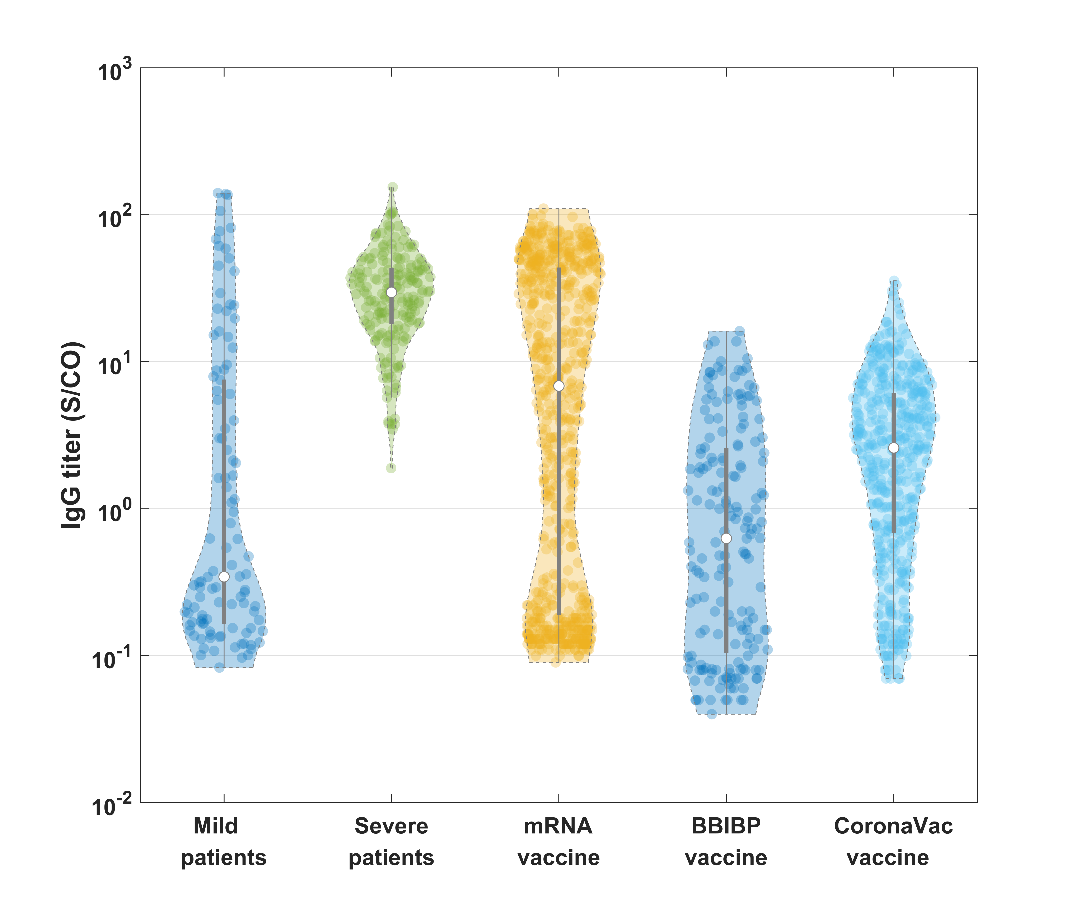


D
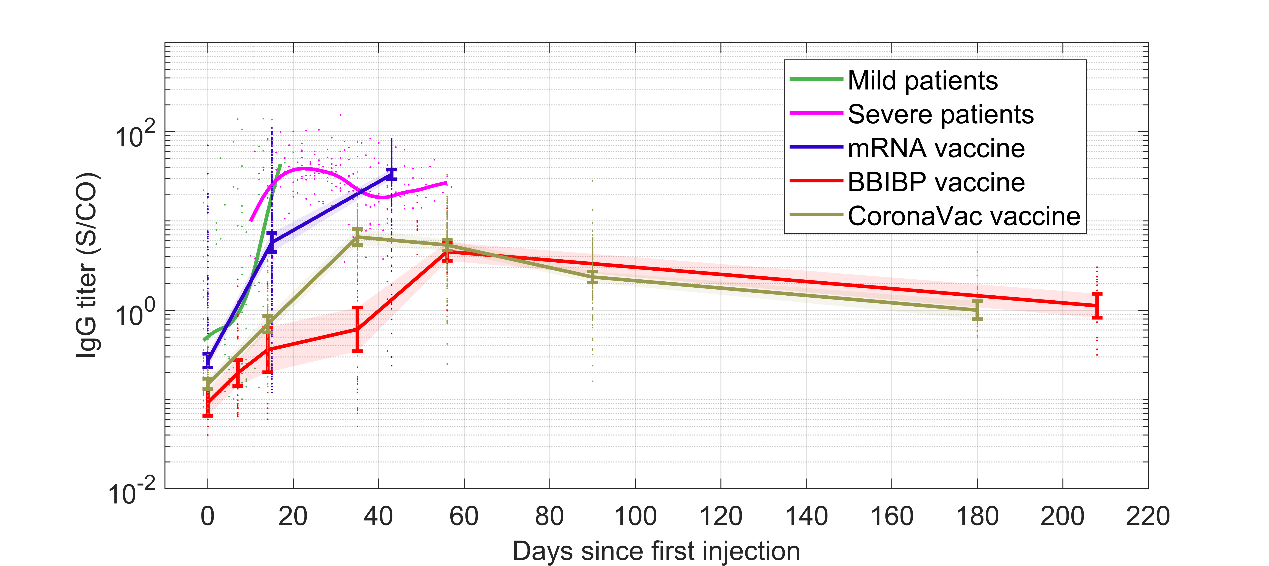

Supplement: Supplementary file 1 [file DataSheet_1.docx]
