# Supplementary material for: Identifying COVID-19 Infections From a Vaccinated Population Using Specific IgA Antibody Test
Source: Front Immunol. 2022 Jan 31;13:821218. doi: 10.3389/fimmu.2022.821218 (PMC8841746; doi:10.3389/fimmu.2022.821218)
Supplement: Supplementary file 2 [file Table_1.docx]

**Table S1. Patient characteristics.**

| **Characteristic** | **Infected Patients** | | **Vaccine Volunteers** | | |
| --- | --- | --- | --- | --- | --- |
|  | Mild | Severe | Pfizer-BioNTech | BBIBP | CoronaVac |
| Numbers | 59 | 14 | 247 | 131 | 103 |
| Sex (male, n (%)) | 31 (52.5) | 11 (78.6) | 51 (20.6) | 50 (38.2) | 26 (25.2) |
| Age (years, mean ± SD) | 43.6 ± 16.8 | 61.1 ± 10.7^*^ | 56.0 ± 20.9 | 33.3 ± 8.3 | 34.8 ± 10.8 |
| With underlying diseases^**^, n (%) | 18 (30.5) | 9 (64.3)^*^ | 0 | 0 | 0 |
| Duration of hospitalization (days, mean ± SD) | 20.6 ± 7.8 | 58.5 ± 7.8^*^ | - | - | - |

* P < 0.05 (Mild VS. Severe group, significance was determined by unpaired Wilcoxon test).

** Underlying diseases include hypertension, diabetes, [coronary](D:/%E8%BD%AF%E4%BB%B6/Youdao/Dict/7.5.2.0/resultui/dict/?keyword=coronary)[heart](D:/%E8%BD%AF%E4%BB%B6/Youdao/Dict/7.5.2.0/resultui/dict/?keyword=heart)[disease](D:/%E8%BD%AF%E4%BB%B6/Youdao/Dict/7.5.2.0/resultui/dict/?keyword=disease) and chronic obstructive pulmonary disease.
